# Screening Fluorescent Voltage Indicators with Spontaneously Spiking HEK Cells

**DOI:** 10.1371/journal.pone.0085221

**Published:** 2013-12-31

**Authors:** Jeehae Park, Christopher A. Werley, Veena Venkatachalam, Joel M. Kralj, Sulayman D. Dib-Hajj, Stephen G. Waxman, Adam E. Cohen

**Affiliations:** 1 Department of Chemistry and Chemical Biology, Harvard University, Cambridge, Massachusetts, United States of America; 2 Department of Neurology, Yale School of Medicine, and Neurorehabilitation Research Center, Veterans Affairs Hospital, West Haven, Connecticut, United States of America; 3 Department of Physics, Harvard University, Cambridge, Massachusetts, United States of America; Brigham & Women's Hospital - Harvard Medical School, United States of America

## Abstract

Development of improved fluorescent voltage indicators is a key challenge in neuroscience, but progress has been hampered by the low throughput of patch-clamp characterization. We introduce a line of non-fluorescent HEK cells that stably express Na_V_ 1.3 and K_IR_ 2.1 and generate spontaneous electrical action potentials. These cells enable rapid, electrode-free screening of speed and sensitivity of voltage sensitive dyes or fluorescent proteins on a standard fluorescence microscope. We screened a small library of mutants of archaerhodopsin 3 (Arch) in spiking HEK cells and identified two mutants with greater voltage-sensitivity than found in previously published Arch voltage indicators.

## Introduction

Improved fluorescent voltage indicators would enhance our ability to study the electrophysiology of neurons, cardiac cells, and other electrically active cell-types, *in vitro* and *in vivo* [1,2]. Complex network behavior, sub-cellular electrical dynamics, and long-term changes in electrophysiological function are all quantities that are difficult or impossible to measure with electrode-based techniques. Non-contact recording of electrical waveforms would also facilitate screens for drugs that modulate neuronal or cardiac function.

An ideal voltage sensor must meet several criteria. Its fluorescence should be bright, photostable, and at a wavelength convenient for biological imaging. It should respond quickly to a step in voltage, and have a large fractional change in fluorescence for physiologically relevant voltage swings (-70 mV to +30 mV in neurons). It should traffic efficiently to the plasma membrane, and its fluorescence and voltage response should not be influenced by other cellular parameters such as pH, Ca^2+^, or composition of the membrane. Illumination should not induce phototoxicity, changes to the membrane potential, or other changes in the cellular physiology. 

**Figure 1:**
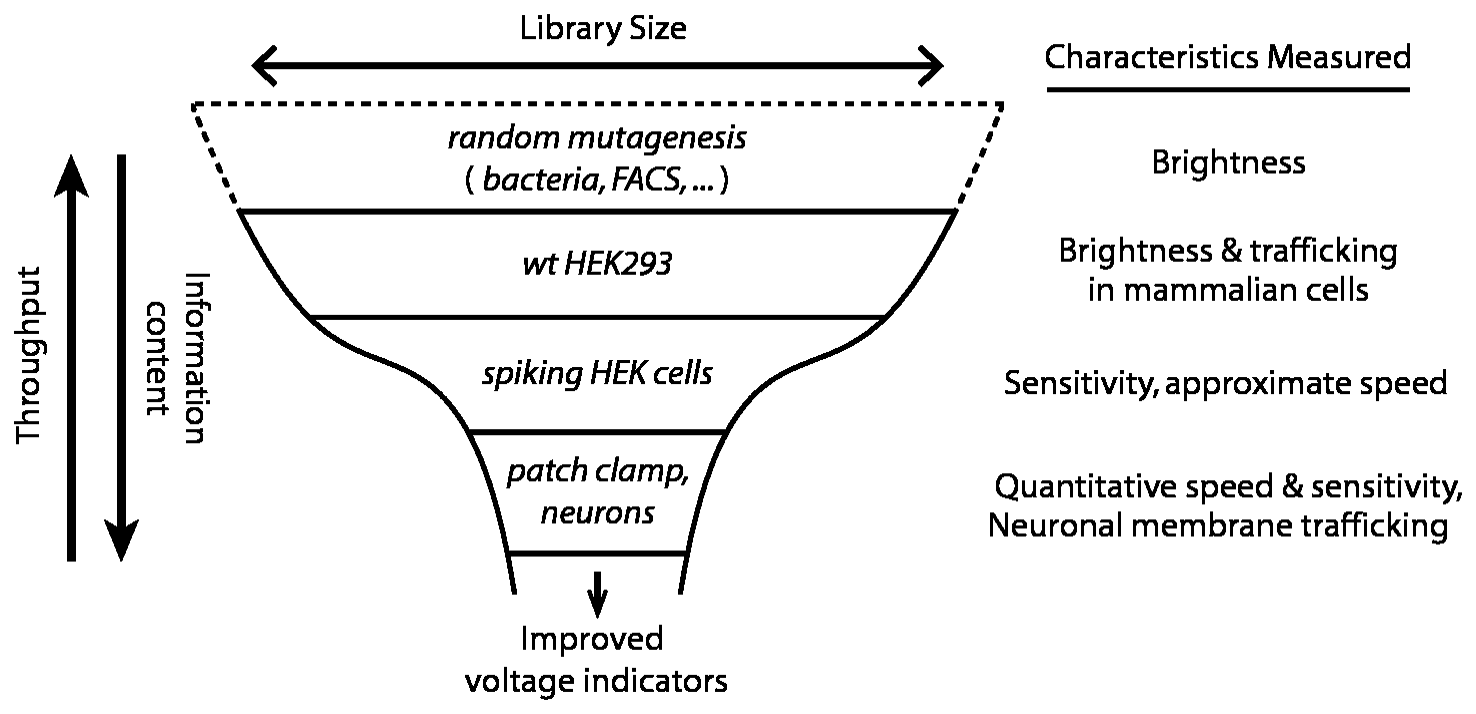
Hierarchical approach to screening for improved voltage indicators. At each level of the screen an increasingly complex measurement is applied to a smaller number of cells. Fluorescence brightness is readily screened in a large library via e.g. bacterial colony screening or fluorescence activated cell sorter (FACS). Membrane trafficking can be assessed via image analysis of expression patterns in HEK cells. Spiking HEK cells provide a critical gate by filtering based on voltage sensitivity, and speed (above 4 ms). Ultimately, fluorescent voltage indicators intended for neuronal use must be characterized by patch clamp measurements in neurons.

Clearly no single screen can measure all of these parameters at once. Rather, the search for improved voltage indicators should proceed hierarchically, with easily measured parameters such as brightness being tested on large numbers of mutants, and more challenging assays such as voltage sensitivity being tested on mutants that have passed simpler selection criteria (Figure 1). In a hierarchical screen, one must also be cautious about translating results between experimental systems. For instance, protein trafficking and folding can be dramatically different in bacteria and eukaryotic cells [3]. Thus one should screen in cells as closely related as possible to the intended application.

Within this hierarchy of screens, tests for voltage sensitivity and speed of response have been particularly challenging. Patch clamp measurements in a fluorescence microscope provide quantitative and precise data, but manual patch clamp is laborious and slow. Transmembrane voltages induced by bath electrodes can activate fluorescent voltage indicators in mammalian cells, but the fluorescence responses are difficult to calibrate because the time course and amplitude of the membrane voltage depend on a cell’s neighbors and morphology in a complex way [4]. When cultured under the right conditions, both neurons [5] and cardiomyocytes [6] generate spontaneous patterns of electrical activity. Ca^2+^ fluxes in neurons induced by field stimulation have been used to screen for improved genetically encoded Ca^2+^ indicators [7], but the cost and logistics of culturing primary cells in large numbers can be limiting. 

A recent report showed that upon stable expression of a small number of ion channels, rapidly growing and easily cultured human embryonic kidney (HEK) 293 cells generated spontaneous action potentials [8]. Those cells, however, could not be used for testing fluorescent voltage indicators because multiple fluorescent markers spanning the visible spectrum were used to select clones expressing the desired ion channels. Here we introduce a transgenic line of HEK 293 cells that generate stereotyped spontaneous electrical spikes and have a dark fluorescence background. Requests for cells should be directed to the corresponding author.

We first applied patch clamp electrophysiology and voltage-sensitive dye (VSD) imaging to characterize the waveform and reproducibility of the spiking behavior, and recorded movies of voltage waves in syncytial monolayers. We then developed assays to use the spiking HEK cells to test VSDs and genetically encoded voltage indicators (GEVIs) and validated the assays with well-characterized reporters of both types. A screen of a small library of Archaerhodopsin 3 (Arch) mutants yielded several mutants with improved sensitivity relative to previously published variants. 

## Results

**Figure 2:**
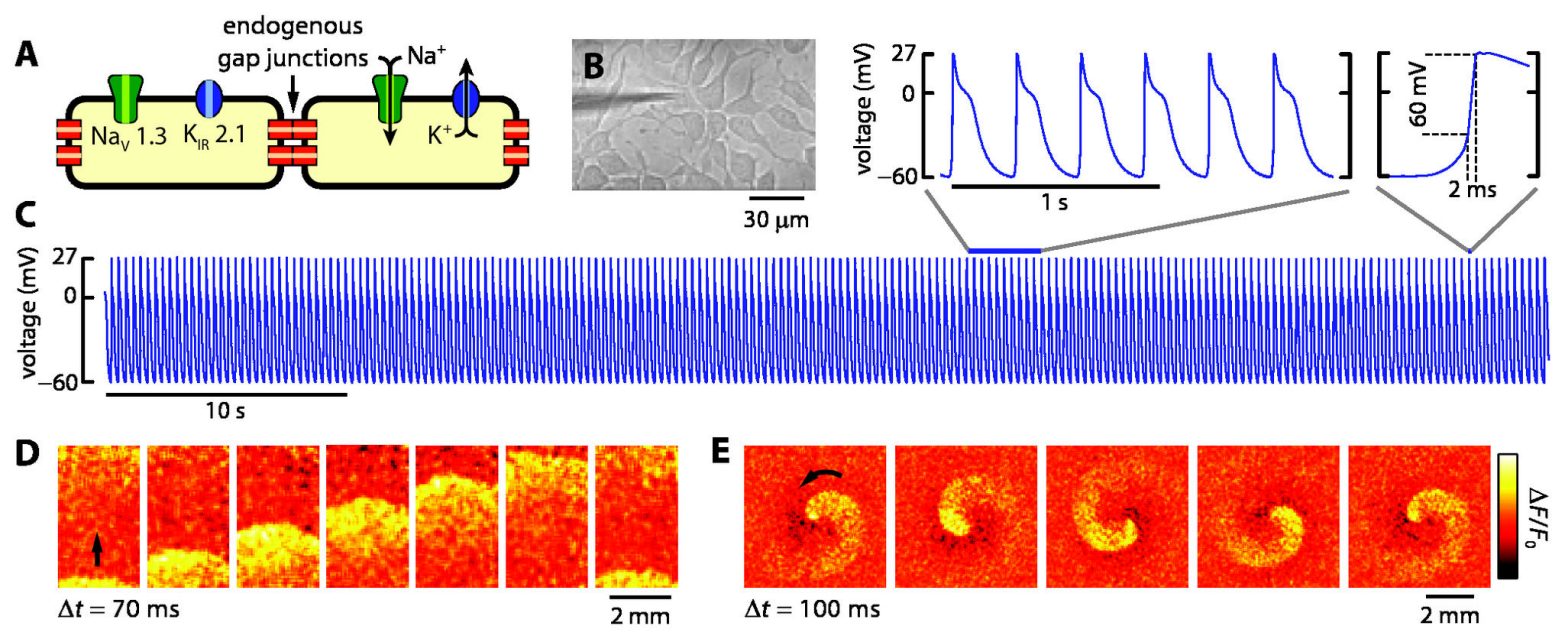
HEK cells expressing Na_V_ 1.3 and K_IR_ 2.1 generate spontaneous electrical spikes. A) Cartoon showing ion channels whose expression is sufficient to induce electrical spiking in a syncytial monolayer. B) Image of spiking HEK cells. A patch pipette is also visible. C) Patch clamp recording of membrane voltage in a single spiking HEK cell. D) Voltage-sensitive dye images showing electrical wave propagation in a culture of spiking HEK cells. E) Waves originated as self-reinforcing spirals. Videos S1-S4 show more propagation patterns in spiking HEK cells.

We made easily cultured excitable cells by generating a clonal line of HEK 293 cells stably expressing the voltage-gated sodium channel Na_V_ 1.3 and the inward rectifying potassium channel K_IR_ 2.1 (Figure 2A). Na_V_ 1.3 was selected because it produces an inward current in response to small depolarization above resting potential, and rapidly recovers from inactivation and thus can sustain repetitive firing [9]. K_IR_ 2.1 was selected because it produces a stable resting voltage near the K^+^ reversal potential and determines the resting potential in many excitable cell types [10]. Additionally, K_IR_ 2.1 closes upon depolarization, producing action potentials of sufficient duration to propagate robustly and produce regenerative oscillations, even in cell cultures with weak gap junction coupling. 

To avoid fluorescent background from expression markers, stable inserts were selected using antibiotic resistance (*Methods* and Refs. 9,11) followed by manual patch clamp to identify clones with robust sodium and inward rectifier potassium currents. Cells expressing Na_V_ 1.3 alone showed rapidly inactivating inward currents upon a depolarizing step from -70 mV to 0 mV, but had resting potentials between -10 and -20 mV. Upon antibiotic selection for expression of K_IR_ 2.1, isolated cells had resting potentials between -50 and -70 mV. A single clone was expanded for detailed characterization.

**Figure 3:**
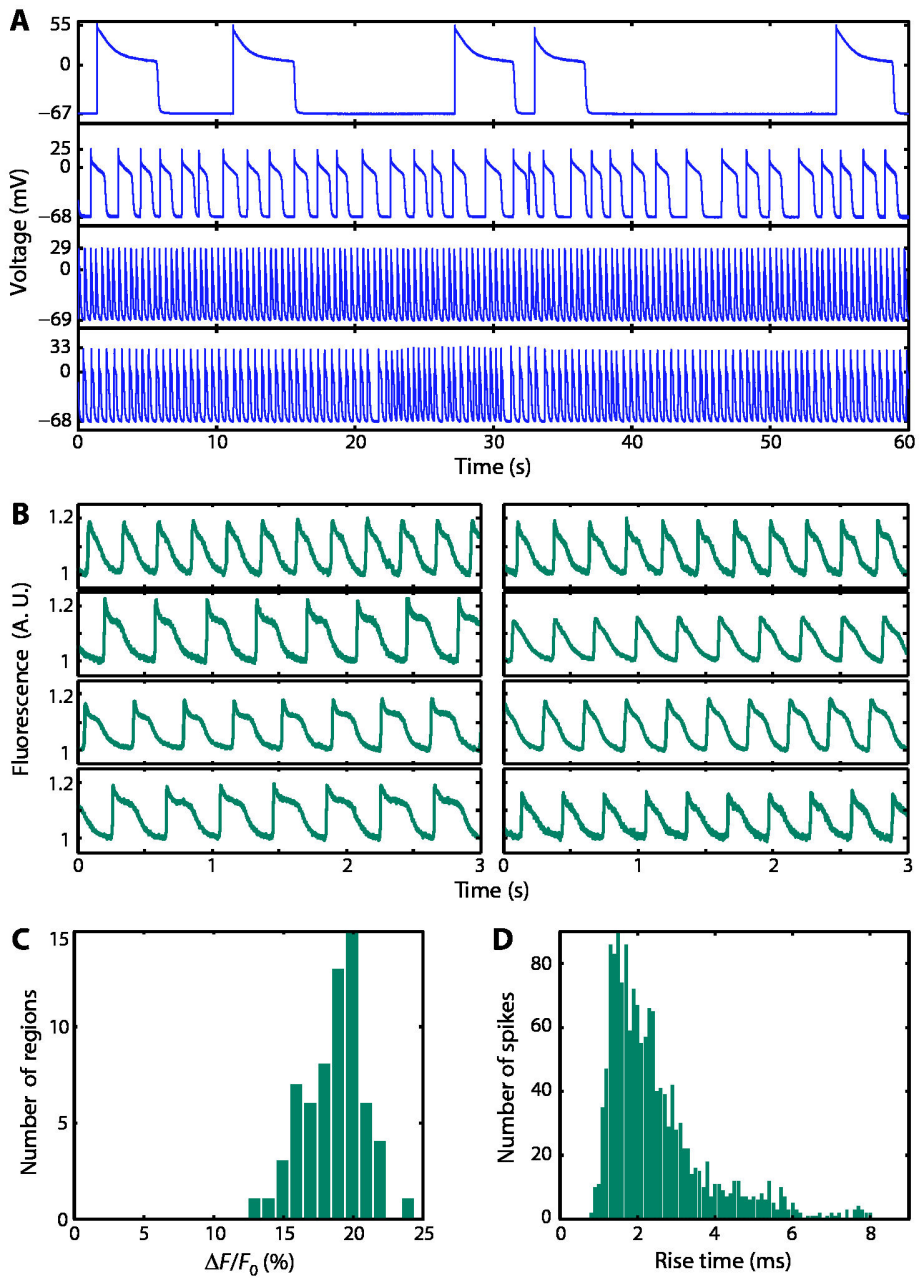
Reproducibility of spiking. A) Patch clamp voltage recordings of spontaneously spiking HEK cells at different levels of confluence. Although the beat rate varied with cell density, the rising edge was consistently fast (2-3 ms) and the variation in the voltage swing was typically ~10% between dishes. B) Representative fluorescence traces from eight different dishes treated with the voltage sensitive dye VF2.1.Cl. Fluorescence was recorded at a 1 kHz frame rate. C) Histogram of fluorescent spike amplitudes. D) Histogram of spike rise time (40% to 70% depolarization) as recorded optically. Histograms show the aggregate results from 13 dishes of cells and 15 locations within each dish.

When these cells were grown into syncytial monolayers at 80 - 95% confluence (e.g. Figure 2B), patch clamp measurements reported spontaneous electrical spikes at a frequency of ~3 Hz (Figure 2C). All patch clamp and optical measurements were performed at room temperature. Although the beat rate varied with cell density and culture conditions (Figure 3A), the overall voltage swing and rise time were consistent. The resting voltage was –66 ± 5 mV and peak depolarization was +34 ± 12 mV, with a 3 ± 2 ms rise between -35 and +15 mV (n = 10 cells; mean ± s.d.). This spontaneous spiking persisted for 2 - 3 days before the culture became overgrown. Spiking could be arrested by blocking sodium channels with 10 nM tetrodotoxin. We attribute the negative resting voltage to the potassium channels and the rapidly inactivating depolarizing current to the sodium channels. Intercellular electrical coupling was likely mediated by connexin 45 gap junctions which are endogenously expressed in HEK cells [12,13]. 

To map the spatiotemporal pattern of spiking we stained dishes with the fast and sensitive VSD VF2.1.Cl [14]. Imaging the VSD with a custom wide-field microscope at low magnification (*Methods*) showed propagating waves (Figure 2D and Video S1) with a typical velocity of 2 cm/s that emanated from spiral sources (Figure 2E and Video S2). The centers of the spirals did not appear associated with morphological defects (Video S3) and indeed spiral centers drifted over time. We occasionally observed chaotic regions at the boundary between colliding waves (Video S4). These patterns are consistent with models of wave propagation in excitable media [15,16]. Self-reinforcing spiral waves, which are a stable state of excitable media, explain periodic electrical beating in the absence of pacemaker cells and explain why spontaneous spiking is only observed in electrically coupled monolayers. 

While visually striking at low magnification (≤ 10 x magnification), the wave nature of electrical propagation was inconsequential for fluorescence measurements taken at single-cell resolution (60 x magnification). The propagating wavefront took ~1 ms to cross a cell, while the rise time of the voltage was ~3 ms. Thus the voltage across a single cell was effectively uniform, and the temporal resolution was not degraded when optical measurements were restricted to individual cells.

To quantify the reproducibility of the spiking waveform in dishes with carefully controlled cell density, we made single-cell optical recordings from 13 dishes and 15 locations within each dish, using the VSD VF2.1.Cl. Representative time traces and summary statistics are shown in Figure 3B-D. The mean Δ*F*/*F* across all measurements was 19.2 ± 2.1% (n = 195 measurements, mean ± s. d.). The VSD measurements and patch clamp measurements reported similar fractional variation in spike amplitude, indicating that both measurements were of comparable precision. Thus by imaging a candidate fluorescent voltage indicator dye in spiking HEK cells, one can determine sensitivity with a precision of ~10%. 

To determine the temporal resolution of the spiking HEK cell assay, we measured the speed of the action potential upswing using the VSD VF2.1.Cl. 96% of cells had a rise time (40% to 70% depolarization) of less than 8 ms; of these the mean rise time was 2.5 ms ± 1.3 ms (n = 1531 beats). The 40% and 70% thresholds were selected to bracket the fastest part of the rising edge, and thereby to maximize the temporal resolution to the assay. The apparent response time of an indicator to a positive-going step in voltage was determined by the convolution of the rise time of the voltage (2.5 ms), the exposure time of the camera (1 ms) and the true response time of the indicator. Thus we estimate that response times slower than ~4 ms could be measured. Consistent with this estimate, wild-type Arch had an apparent response time in spiking HEK cells of 4 ms (see below), while previous patch-clamp measurements showed an underlying response time of 0.6 ms [17]. Although it will be difficult for our assay to quantify the multi-exponential kinetics observed in the step response of some voltage indicators, the apparent time constant is an effective measure for comparing the relative performance of different sensors.

Neuronal action potentials are typically ~1 ms in duration, so the spiking HEK cells do not provide absolute confirmation that an indicator is fast enough for neuronal recording. Furthermore, we found that the repolarization rate of the HEK action potential was variable between cultures, so we did not use the spiking HEK cells to measure off-rates. Nonetheless, the 4 ms time resolution on the rising edge of the spiking HEK cells is faster than most genetically encoded voltage indicators reported to date. Thus the spiking HEK assay provides a stringent gate that significantly reduces the number of indicators that must ultimately be tested by manual patch clamp.

**Figure 4:**
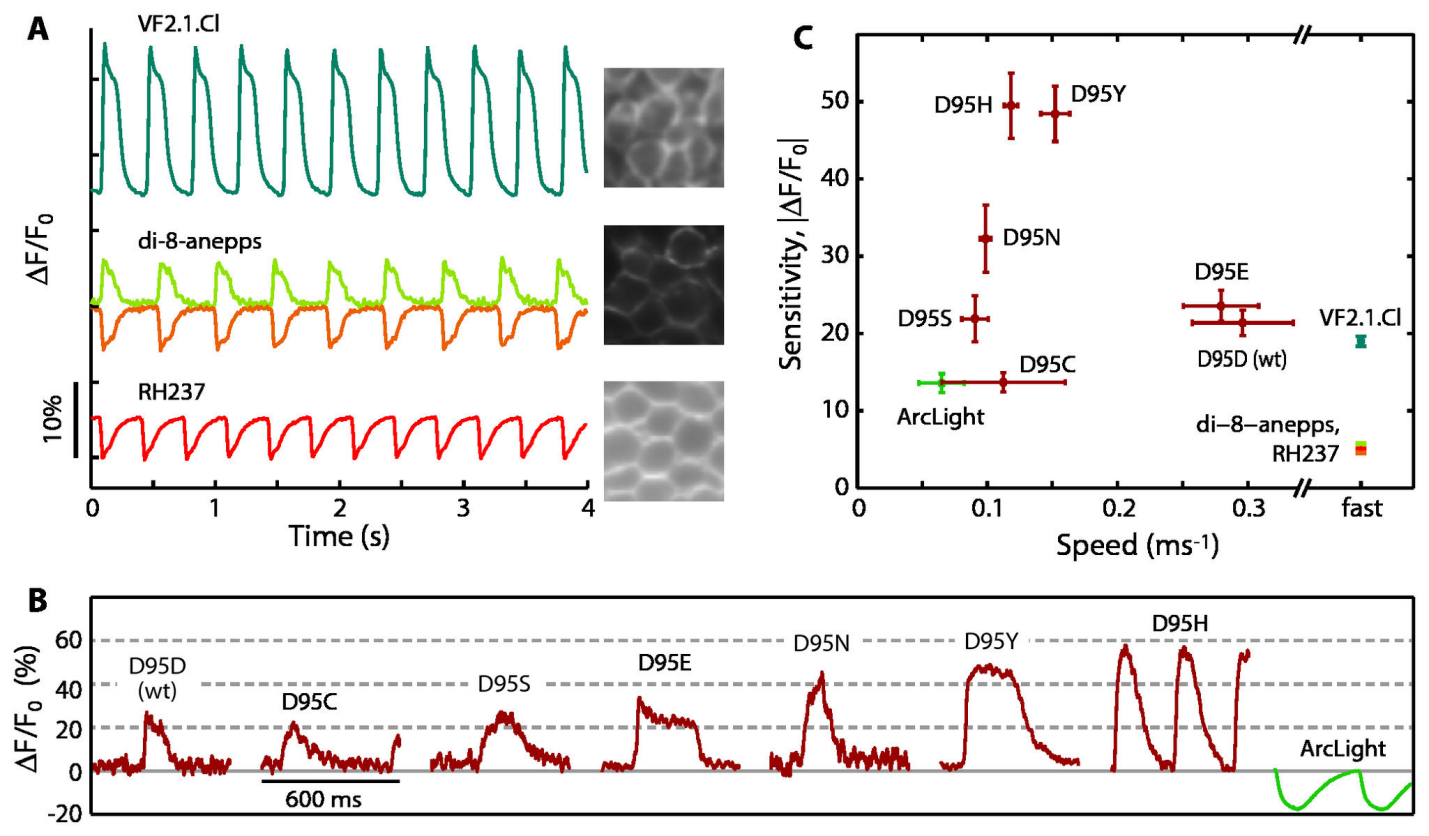
Spiking HEK cells report sensitivity and approximate speed of voltage indicators. A) Voltage-sensitive dyes showed fluorescence sensitive to electrical spikes. For VF2.1.Cl excitation was at 488 nm and emission was collected from 525- 575 nm. For di-8-ANEPPS, excitation was at 488 nm, fluorescence for the positive-going signal was collected from 525-575 nm and negative-going signal was collected between 660 and 740 nm. For RH237 excitation was at 532 nm and emission was 660 - 740 nm. The images (40 µm across) show staining efficiency. B) Representative fluorescence waveforms of genetically encoded voltage indicators. The Arch mutants were excited at 640 nm (~500 W/cm^2^), with fluorescence emission collected from 660-740 nm. ArcLight-A was excited at 488 nm with fluorescence emission collected from 525- 575 nm. C) Sensitivity and speed of fluorescent voltage indicators. The speeds plotted for the genetically encoded reporters represent the apparent speed, determined by convolution of the upswing of the action potential, the camera exposure, and the underlying speed of the reporter. Thus the apparent speeds of Arch(D95E) and Arch WT were slower than their true speeds. The VSDs are known to be significantly faster than 4 ms, so no effort was made to measure their speeds optically. The sign of response of ArcLight-A, RH237, and the negative-going signal of di-8-ANEPPS have been inverted to facilitate comparison. Error bars represent s.e.m. of n = 7 - 38 single-cell measurements.

We tested several other VSDs for sensitivity (Figure 4A) to further calibrate the ability of the spiking HEK cells to reproduce known sensitivity parameters. These measurements reproduced the known attributes of the dyes, including the voltage-dependent spectral shift of di-8-ANEPPS [18] and the inverse voltage-sensitivity of RH237 [19]. Thus spiking HEK cells provide a platform for facile screening of candidate small-molecule voltage indicators.

We then tested the ability of spiking HEK cells to screen protein-based sensors. We developed a protocol to express and image GEVIs in spiking HEK cells (*Methods*) and tested ArcLight-A (Q239) [20], Arch and Arch(D95N) [21], and the 18 previously untested Arch(D95X) mutants. Of these new mutants, five showed voltage-sensitive fluorescence. Traces from representative spikes are shown in Figure 4B. By analyzing spike trains from many cells, one can measure sensitivity and speed for each mutant. The sensitivity is given by (F_max_ - F_min_)/F_min_. Speed measurements can be distorted by convolution of the intrinsic sensor step response with the underlying waveform of the spiking HEK action potential. Thus multi-exponential response kinetics, as previously observed in Arch(D95N) [17] and ArcLight [20], were not resolved. However, fitting the rising edge to a single exponential (see *Methods*) yielded an effective time constant τ that was indicative of the relative speed of different mutants. Figure 4B shows a scatter plot of speed (1/τ) versus sensitivity. The ideal fast and sensitive indicator would lie in the upper right corner of the graph. The most promising mutants identified in the screen were Arch D95H, D95Y, and D95E.

**Figure 5:**
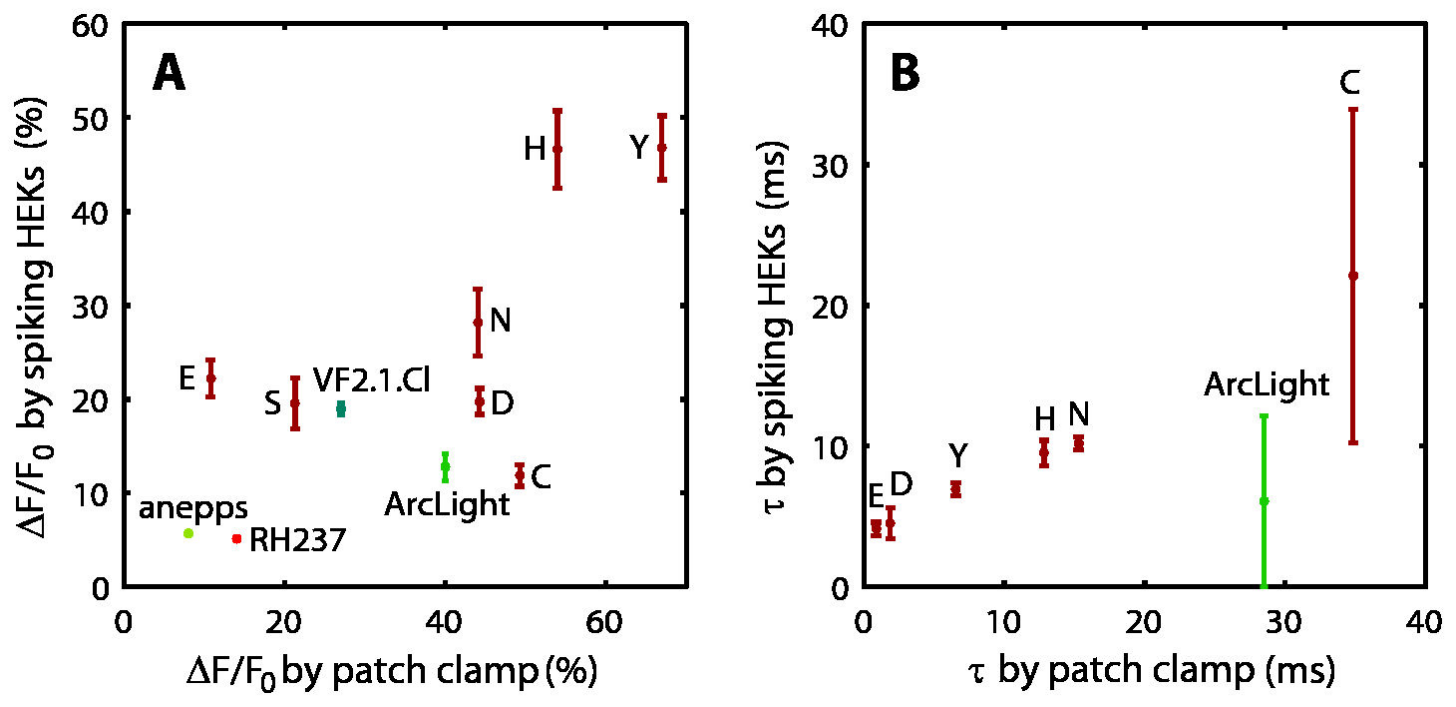
Spiking HEK sensitivity and speed correlate with patch clamp results. A) Sensitivity per 100 mV (-70 mV to +30 mV) as measured by manual patch clamp and spiking HEKs. Literature values are used for RH237 [19], ArcLight-A (Q239) [20], VF2.1.Cl [14], and di-8-ANEPPS [14]. B) Effective rising edge time constants measured with patch clamp and with spiking HEKs. For patch clamp data, the time constant is a weighted average of the time constants from a bi-exponential fit.

We compared the speed and sensitivity of reporters as measured in spiking HEK cells with corresponding measurements from whole-cell voltage clamp in regular HEK cells (Figure 5). Sensitivities for the VSDs and ArcLight were drawn from the literature. Sensitivity correlated positively with patch-clamp results, with an *r* ^2^ value of 0.50 and speed correlated positively with patch-clamp results with an *r* ^2^ value of 0.57, demonstrating that data from spiking HEK cells can be used to rank sensor performance. The apparent sensitivity of slower voltage reporters such as ArcLight and Arch(D95C) was reduced because the sensors did not reach maximum response within the duration of the depolarized phase of the action potential. Convolution of the action potential waveform with the sensor impulse response as measured by patch clamp predicts a nearly 2-fold reduction in the apparent sensitivity for these two mutants, consistent with Figure 5A.

**Figure 6:**
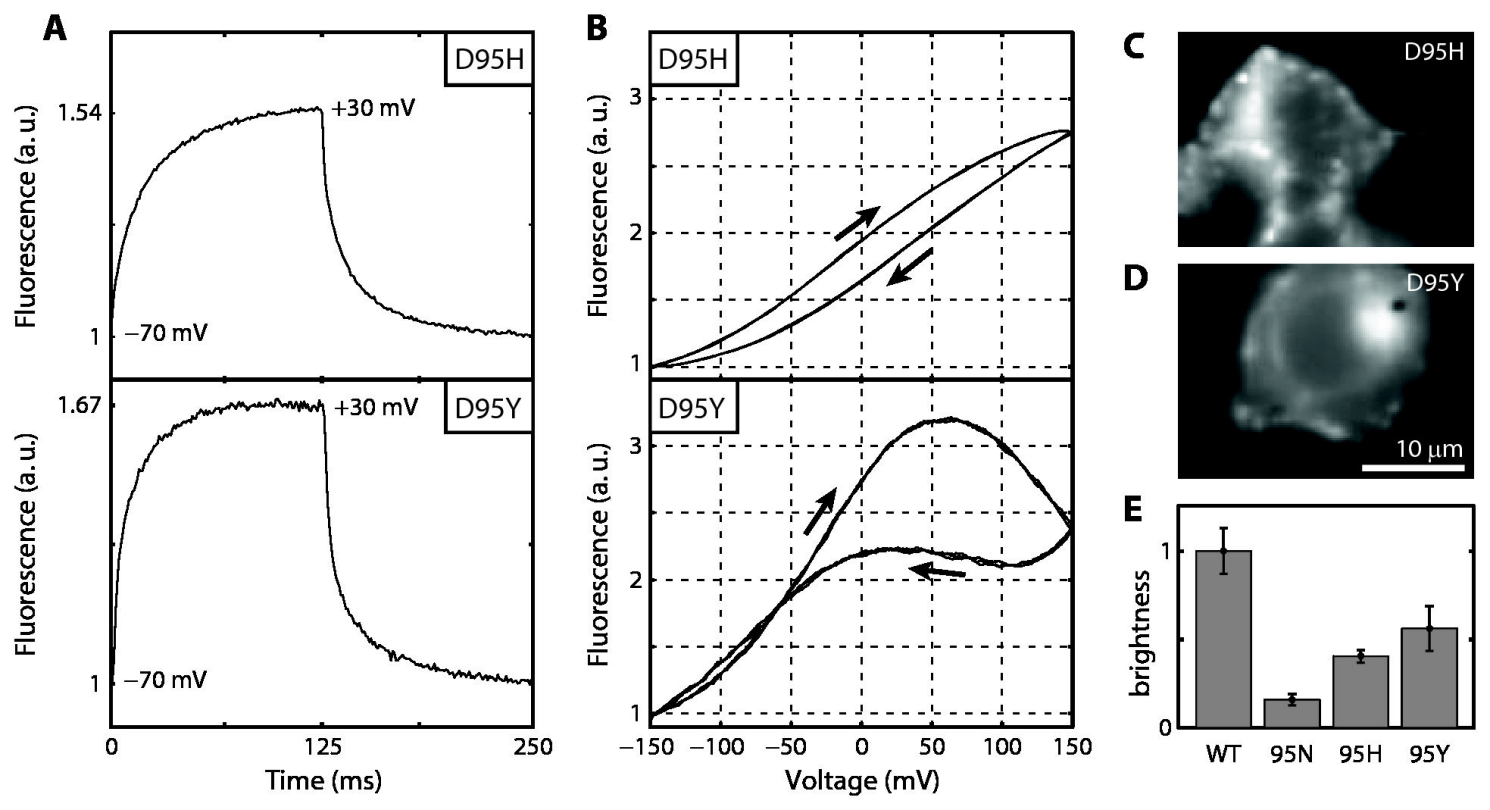
Patch clamp characterization of Arch(D95Y) and D95H. A) Fluorescence response to a step in membrane voltage from -70 to +30 mV. Each trace is the average of 38 steps. Fits to a bi-exponential are shown in Table 1. B) Fluorescence as a function of membrane voltage. Both proteins showed hysteresis at slow sweep speeds (0.5 Hz), indicating multiple stable states. Each trace shows the fluorescence as the voltage was cycled three times in the direction indicated by the arrows. Raw fluorescence was corrected for photobleaching of the baseline. C) and D) Images of Arch fluorescence in the cells measured in A and B. E) The relative brightness, defined as the ratio of Arch fluorescence to fluorescence from a covalently bound GFP, for key Arch variants at a 640 nm illumination intensity of 440 W/cm^2^. The brightness for each mutant was normalized to WT brightness; error bars show SEM from ~15 cells per mutant.

Patch clamp and brightness measurements are shown in Figure 6 for the most promising mutants Arch(D95Y) and D95H. Like the previously reported mutant Arch(D95N), but in contrast to Arch WT, neither showed a photocurrent. The response speed was probed with a square wave (Figure 6A). The rising edge was fit by a double-exponential of the form:

$F(t)=1+A[1-\exp(-t/\tau_{1})]+B[1-\exp(-t/\tau_{2})]$

and the falling edge was fit by a function of the form:

$F(t)=1+A\exp(-t/\tau_{1})+B\exp(-t/\tau_{2})$

The fitting parameters are collated in Table 1, which reveals that the fast component comprises a significantly larger fraction of the voltage response in Arch(D95Y) than in D95N. Sensitivities are shown in Figure 6B, and again D95Y considerably outperforms D95N. Arch(D95Y) showed an unusual hysteresis in the plot of fluorescence vs. voltage, distinct from photobleaching. This hysteresis did not manifest when the voltage was limited to < +50 mV, and thus is not relevant for biological imaging. However the hysteresis is interesting from a photophysical perspective: the non-monotonic and history-dependent fluorescence suggest that the protein has multiple voltage-dependent rates in its photocycle. The brightness for both D95H and D95Y (Figure 6E), calculated by taking the ratio of Arch fluorescence to GFP fluorescence and averaging over many cells, lay between the brightness for Arch WT and D95N. Arch(D95Y) showed improved sensitivity, speed, and brightness relative to the previously reported non-pumping Arch(D95N) and is a promising candidate for use as genetically encoded fluorescent voltage indicator. However, we would not recommend immediate adoption of Arch(D95Y) as further-improved Arch mutants will soon be published.

**Table 1:** Step response fitting parameters from patch clamp characterization.

		***τ*_*1*_ (ms)**		***τ*_*2*_ (ms)**	
Arch(D95H) rising	0.34	5	0.66	31	0.54
Arch(D95H) falling	0.40	2	0.60	23	0.51
Arch(D95Y) rising	0.44	3	0.56	20	0.66
Arch(D95Y) falling	0.61	4	0.39	28	0.69
Arch(D95N) rising	0.17	1	0.83	20	0.39
Arch(D95N) falling	0.22	3	0.78	20	0.38

## Discussion

Our screen of a small GEVI library revealed several important considerations when making measurements with spiking HEK cells. First, the fluorescence trace is the convolution of the electrical waveform with the sensor impulse response. This fact restricts quantification of response times to values between ~4 and ~50 ms. The assay accurately reports sensitivity in fast sensors, but it underestimates sensitivity of slow sensors, as it did for ArcLight. Second, as with any genetically encoded reporter, one must adjust transfection level and expression time to identify a window where expression is high enough to produce a robust signal, but not so high that it perturbs the cell. Expression of candidate reporters at high levels might reduce the amplitude of voltage swings in spiking HEKs relative to non-expressing cells, and we have detected some transfected cells with significantly reduced sensitivity. As a result, the maximum measured sensitivity, instead of the mean, is likely a better indicator of sensor performance. The scatter plots in Figures 4C and 5A show mean values and so underestimates actual indicator sensitivities. In light of these considerations, measurements in spiking HEKs should not be used as a substitute for quantitative patch-clamp methods, but instead to rapidly identify mutants that show high likelihood of being fast and sensitive. 

Spiking HEK cells can facilitate tests of any type of voltage indicator. Here we tested VSDs and GEVIs, but the same tools can be applied to hybrid small molecule-protein voltage sensors [22] and to recently proposed quantum dot-based sensors [23,24]. The voltage oscillations in spiking HEK cells may also prove useful in testing for compounds that modulate ion channel activity: any compound that acts on the sodium channel, potassium channel, or other endogenous or expressed channels will affect the spiking waveform and propagation; and these changes are readily detected via optical imaging with a dye- or protein-based indicator. For instance, Na_V_ 1.3 contributes to neuropathic pain [25], and is thus a target for small molecule drug discovery.

## Methods

### Generation of spiking HEK cells

HEK cells stably expressing Na_V_ 1.3 were obtained from the laboratory of Stephen Waxman [9,11] and grown in a 1:1 mixture of Dulbecco’s Modified Eagle Medium and F-12 supplement (DMEM/F12). This medium contained 10% fetal bovine serum, penicillin (100 U/mL), streptomycin (100 μg/mL), and geneticin (500 μg/mL).

K_IR_ 2.1 was amplified from Addgene plasmid 32669 (pENTR-L5-Kir2.1-mCherry-L2) using primer FWD_BamHI_Kir2.1 (CAT TAG TCT AGA GGA TCC GCC ACC ATG CCA ACT TTG TAT ACA AAA GTT GCC GC) and REV_Kir2.1_SalI (CTA ATG GTC GAC TCA TAT CTC CGA TTC TCG CCT TAA GGG C). The PCR product was cloned into a pLenti-CMV-puromycin vector. The resulting vector (pLenti-CMV-Kir2.1-puromycin) was inserted into lentivirus for infecting Na_V_ 1.3 HEK cells. After 24 hrs of virus exposure, puromycin was added to a final concentration of 2 μg/mL. Cells were cultured for 14 days and then single cells were dispersed in wells of a 96 well plate. Monoclonal lines were screened using by patch clamp electrophysiology to detect cells with a resting membrane potential < -55 mV and the ability to generate action potentials upon a depolarizing current pulse. In the selected Na_V_ 1.3/K_IR_ 2.1 clonal line, the resting potential was -66 mV, compared to -20 mV in wild-type HEK cells.

### Growth conditions for spontaneous spiking

A single monoclonal line was cultured in DMEM/F12, 10% FBS, 1% penicillin (100 U/mL), streptomycin (100 μg/ml), geneticin (500 μg/mL) and puromycin (2 μg/mL). Single-cell patch clamp measurements were performed at, 10-20% confluence. Spontaneously generated action potentials began at ~80% confluence. For imaging, cells were grown on a coverglass-bottom dish (P35G-1.5-14-C MatTek), which was pre-treated with Matrigel (BD Biosciences) in a 1:50 dilution in DMEM for 30 min at 37° C. 

### Electrophysiology

At the time of imaging, culture medium was replaced with Tyrode’s solution containing, in mM, 125 NaCl, 2 KCl, 3 CaCl_2_, 1 MgCl_2_, 10 HEPES, 30 glucose (pH 7.3) and adjusted to 305–310 mOsm with sucrose. Glass micropipettes had 7-12 MΩ tip resistance and were loaded with internal solution (in mM 125 potassium gluconate, 8 NaCl, 0.6 MgCl_2_, 0.1 CaCl_2_, 1 EGTA, 10 HEPES, 4 Mg-ATP, 0.4 Na-GTP (pH 7.3); adjusted to 295 mOsm with sucrose). A Sutter MP285 manipulator was used for pipet positioning and an Axopatch 200B amplifier (Molecular Devices) was used for whole-cell patch clamping. Data was acquired using a National Instruments DAQ card (PCIe-6343) controlled via home-made software written in LabView. Fluorescence measurements during patch clamp recording were made on a home-built fluorescence microscope using an electron multiplying charge coupled device (EMCCD; Andor iXon3 860). The light was collected with a 60x oil immersion objective (NA = 1.49, Olympus APON60XOTIRF) and optical recordings were performed at a sampling rate of 1 kHz.

### Dye-loading procedure

VSDs were dissolved in DMSO (stock concentration in parenthesis) and used with final concentrations as following: 0.2 μM VF2.1.Cl (200 μM), 2 μM di-8-ANEPPS (2 mM), 5 μM RH237 (10 mM). Cells were incubated with the VSD in Tyrode’s solution at room temperature for 10 minutes, then washed with dye-free Tyrode’s solution followed by imaging. 

### Generation of Arch(D95X) mutants

A library of Arch(D95X) mutants was generated by performing saturation mutagenesis of residue Asp95 in Archaerhodopsin-3 in the pET-28b vector using the primers D95X_FWD (5’-CAGGTACGCCNNKTGGCTGTTTACCACCCCACTTCTG) and D95X_REV (5’-GTAAACAGCCAMNNGGCGTACCTGGCATAATAGATATCCAACATTTCG). The 25 µL saturation mutagenesis reaction contained: 50 ng template DNA (WT Arch in pET-28b); 60 nM of each primer (D95X_FWD and D95X_REV); 0.5 μL *PfuUltra* high-fidelity DNA polymerase (Stratagene); 2.5 μL of 10x *PfuUltra* buffer (Stratagene); and 300 µM dNTPs. The reaction conditions were: (1) 95 °C for 5 minutes; (2) 95 °C for 45 seconds; (3) 53 °C for 50 seconds; (4) 72 °C for 10 minutes; (5) repeat steps 2-4 24 times; (6) 72 °C for 10 minutes. To allow for expression in mammalian cells, the Arch(D95X) library was moved (using Gibson Assembly, New England Biolabs) into a lentiviral mammalian expression vector (Addgene plasmid 22051 cut with the restriction enzymes BamHI and AgeI). The final library consisted of Arch(D95X) fused to C-terminal eGFP, under a ubiquitin promoter.

### Transfection protocol

For GEVI transfection, cells were grown on a Matrigel-treated glass-bottom dish (as described above) until 50-60% confluence. To reduce the number of GEVI plasmids taken up by each transfected cell, the GEVI plasmid was diluted at a 1:200 ratio with pUC19, a plasmid without mammalian promoters. Cells were transfected with a total DNA concentration of 1 μg per 35 mm dish. Transfection was performed using *Trans*IT-293 Transfection Reagent (Mirus Bio) following manufacturer’s instruction. 4 hrs after transfection, the transfection mixture was replaced with growth medium. Cells were imaged 48 hrs post transfection. Prior to imaging, cells were supplemented with 5 μM all-trans retinal for 30 min. to ensure that all Arch proteins contained bound chromophore. Imaging was performed in retinal-free Tyrodes’s solution.

### Video collection

Videos were collected on a home-built epifluorescence microscope using Olympus apochromatic objectives. A UPLSAPO 4X objective (NA = 0.16) was used for Videos S1, S2, & S4 and the water immersion objective UPLSAPO 60XW (NA = 1.2) was used for Video S3. The tube lens focal length was 100 mm, so that the entire field of view of the objectives was imaged onto the detector chip of a Hamamatsu ORCA-Flash 4.0 scientific-CMOS camera. This configuration captured the entire field of view (6 mm square for the 4x objective and 400 μm square for the 60x objective) at diffraction-limited resolution at 100 Hz. The VSD VF2.1.Cl was excited with a 488 nm laser and fluorescence was collected through a 510 nm longpass filter.

### Video analysis

Videos S1, S2, and S4 were generated by subtracting time-averaged fluorescence from each frame in the movie, and scaling the brightness and contrast to highlight the wave. For Video S3, voltage-induced fluorescence change was calculated as for the other videos. Fluorescence changes were displayed on a red to yellow colormap overlaid on the time-averaged fluorescence image. The voltage-dependent fluorescence images were weighted by the time-averaged image to emphasize pixels with a high signal-to-noise ratio.

### Single-cell data

Fluorescence intensities were extracted from single cells using a variant of the method described in [21]. The pixel-by-pixel cross-correlation of fluorescence with whole-cell brightness was used to identify voltage-sensitive pixels. A threshold was applied to this map to select the pixels corresponding to membrane-localized voltage indicator. Fluorescence was determined from these pixels. All movies were analyzed using the same parameters with no user intervention. The same analysis was applied to the spiking HEK data and the patch clamp measurements on Arch(D95Y) and D95H.

The speed of the indicators was estimated from spiking HEK data as follows. The rising edge of each action potential was identified by convolving each fluorescence trace with a step function. All action potentials from a single cell were registered in time and averaged. A single-exponential fit was applied to the rising edge of fluorescence trace. Rate constants from multiple cells were averaged.

## Supporting Information

Video S1 **Plane wave propagation.** A video of voltage recorded using the voltage sensitive dye VF2.1.Cl showing a plane wave, the most common electrical propagation pattern. Brightness is proportional to voltage-induced change in fluorescence. The field of view is 3x6 mm and the playback is slowed 5-fold from real-time.
(AVI)

Video S2 **Stable spiral wave.** A spiral wave which serves as a stable source for periodic electrical spiking. The field of view is 6x6 mm and the playback is slowed 5-fold from real-time.
(AVI)

Video S3 **Spiral wave center.** A magnified view of the center of the spiral wave in video 2. The fluorescence change is plotted in color on a dark red (low voltage) to yellow (high voltage) color map. The induced fluorescence change is overlaid on a grayscale image of the average fluorescence level. The field of view is 400x400 μm and the playback is slowed 5-fold from real-time.
(AVI)

Video S4 **Colliding plane and spiral waves.** A plane wave colliding with a spiral wave showing the annihilation of co-incident waves, as expected for excitable media. The field of view is 5x4 mm and the playback is slowed 5-fold from real-time.
(AVI)
